# Religious Coping and Quality of Life Among Black and White Men With Prostate Cancer

**DOI:** 10.1177/1073274820936288

**Published:** 2020-07-08

**Authors:** Marino A. Bruce, Janice V. Bowie, Haley Barge, Bettina M. Beech, Thomas A. LaVeist, Daniel L. Howard, Roland J. Thorpe

**Affiliations:** 1 Program for Research on Faith, Justice, and Health, Department of Population Health Science, John D. Bower School of Population Health, University of Mississippi Medical Center, Jackson, MS, USA; 2 Hopkins Center for Health Disparities Solutions, Department of Health Behavior and Society, Johns Hopkins Bloomberg School of Public Health, Baltimore, MD, USA; 3 Franklin and Marshall University, Lancaster, PA, USA; 4 Program for Research on Men’s Health, John Hopkins Bloomberg School of Public Health, Baltimore, MD, USA; 5 Department of Health Systems and Population Health Sciences, University of Houston College of Medicine, University of Houston, Houston, TX, USA; 6 Tulane University School of Public Health, New Orleans, LA, USA; 7 Department of Psychological and Brain Sciences, Diversity Science Research Cluster, Texas A&M University, College Station, TX, USA

**Keywords:** prostate cancer, quality of life, health disparities, men’s health, religiosity, spirituality, survivorship

## Abstract

Prostate cancer is a significant impediment in men’s lives as this condition often exacerbates stress and reduces quality of life. Faith can be a resource through which men cope with health crises; however, few studies examine how religion or spirituality can have implications for racial disparities in health outcomes among men. The purpose of this study is to assess the associations between religious coping and quality of life among black and white men with prostate cancer. Data for this investigation were drawn from the Diagnosis and Decisions in Prostate Cancer Treatment Outcomes Study that consisted of 624 black and white men with complete information on the primary outcome and predictor variables. The primary outcome for this study was overall quality of life as measured by the Functional Assessment of Cancer Therapy-Prostate questionnaire. The main independent variable was religious coping measured by 2 subscales capturing positive and negative forms of coping. Black men in the study had lower overall quality of life scores (134.6 ± 19.6) than their white peers (139.8 ± 14.1). Black men in the sample also had higher average positive religious coping scores (12.9 ± 3.3) than white men (10.3 ± 4.5). Fully adjusted linear regression models of the total sample produced results indicating that positive religious coping was correlated with an increase in quality of life (β = .38, standard error [SE] = 0.18, *P* < .05). Negative religious coping was associated with a reduction in quality of life (β = −1.48, SE = 0.40, *P* < .001). Faith-oriented beliefs or perceptions can have implications for quality of life among men with prostate cancer. Sensitivity to the role of religion, spirituality, and faith should be seen by providers of health care as potential opportunities for improved outcomes in patients with prostate cancer and survivors.

## Introduction

Prostate cancer is a slow-growing cancer that is common among men in the United States.^1,2^ Projections for 2019 indicate that 174 650 individuals will be diagnosed with prostate cancer and 31 620 deaths will be associated with this disease.^3^ These statistics are consistent with downward trends in prostate cancer incidence and mortality that have been linked to improvements in screening and treatment.^4^ However, the burden of prostate cancer is not distributed equally across men in the United States, with black men having the highest prostate cancer incidence and mortality rates of any racial and ethnic group.^5^ Recent data indicate that the incidence rate for prostate cancer among black men was 76% higher than the corresponding rate for white men.^5^ Further, black men are more likely to experience early onset of prostate cancer, and twice as likely to die from this disease as their white counterparts.^5-9^

The direct health impact of prostate cancer remains significant; however, this condition also has considerable implications for the overall mental, physical, and social well-being of men as well as their families. Prostate cancer diagnosis and treatment has been linked to a number of psychological (eg, anxiety, depression), physical (eg, erectile dysfunction, urinary incontinence), and social (eg, loss of social support, financial burden) issues that can adversely impact overall quality of life.^8,10-17^ Research examining the impact of prostate cancer on quality of life has been robust; however, very few studies have produced data regarding the quality of life for African American patients with prostate cancer or survivors.^18^ The dearth of research in this area is puzzling given that black men with prostate cancer experience poorer physiological and psychological health than their white peers.^17-19^ Reducing disparities in these outcomes may be linked to the enhancement of quality of life during and after prostate cancer treatment.

The stress associated with prostate cancer and its negative impact on quality of life can be particularly acute for black men. This population has been oppressed, marginalized, and criminalized like no other group in US history^20,21^ and subject to high levels of social and psychological stress from unfavorable social and economic circumstances emerging from institutional discrimination and unfair treatment.^22-26^ Stress has only been recently implicated as a major determinant of black men’s health^27-30^ and a few studies^13,18,26,31^ have highlighted the need for research examining how this group manages or copes with stressors like prostate cancer or its treatment.

Religious faith has been identified as an important coping strategy in managing stress among black men.^23,26^ Broadly, research on faith has typically operationalized it by 2 distinct factors, religiosity or spirituality, which are often confounded. Religiosity involves behaviors associated with social, doctrinal, and denominational characteristics of an organized religion; whereas spirituality refers to the inner experiences with, awareness of, and connection to the transcendent.^32,33^ Each dimension has been associated with the survival of blacks^34,35^ and, in particular, black men.^26,36,37^ Faith-based institutions have been regarded as sacred and safe places where males are welcomed and given affirmative spiritual messages that serve as the foundation of religious coping resources in the face of stressful social environments and situations.^35,38^ Numerous studies have examined the impact of religious coping on variety a of health outcomes over the past 3 decades^39-41^; yet, remarkably few examine the degree to which faith-oriented resources can reduce racial disparities in outcomes associated with prostate cancer and other chronic diseases.^18,42^ The purpose of this study is to assess the associations between religious coping and quality of life among black and white men with prostate cancer.

## Methods

### Data

Data for this investigation were drawn from the Diagnosis and Decisions in Prostate Cancer Treatment Outcomes Study (DADs), a cross-sectional examination of factors associated with treatment modality selection, disease burden, and quality of life among black and white men with prostate cancer. This study was approved by the institutional review boards of the Johns Hopkins Bloomberg School of Public Health, the US Department of Defense, and North Carolina Central Cancer Registry. Potential study participants who were at least 35 years of age, diagnosed and treated for prostate cancer, and classified themselves as black or white were identified from reports generated by a research network of hospitals affiliated with the North Carolina Central Cancer Registry. A rapid case ascertainment procedure, which has been described elsewhere,^12,13,42^ was used to confirm eligibility, and prospective study participants received a DADs study information packet via mail containing a recruitment letter describing the study, a North Carolina Central Cancer Registry brochure, and a copy of the consent forms. The study office phone number was provided in the materials for prospective participants to call for questions or to decline participation. Prospective participants were contacted by phone and study staff confirmed eligibility, introduced and described the study, addressed questions, and asked potential participants to participate in the study. Eligible individuals who agreed to participate completed consent forms and provided verbal consent. After providing consent, the study staff proceeded to administer the questionnaire via interview. The questionnaire comprised of items associated with prostate cancer, its treatment, coping mechanisms, and quality of life during and after treatment. This analysis included those 624 men in the sample who report believing in God and had complete information on quality of life and religious coping variables.

### Study Variables

The primary outcome for this study was overall quality of life as measured by the third version of the Functional Assessment of Cancer Therapy-Prostate (FACT-P) questionnaire.^43^ This instrument contains 12 questions specifically designed for men with prostate cancer along with 34 items from the Functional Assessment of Cancer Therapy-General (FACT-G). The FACT-G has 5 subscales captures the physical, social, emotional, and functional well-being domains of quality of life as well the respondents’ relationship with his physician. Each question has Likert-type responses ranging from 0 to 5. Each item is summed to produce an overall quality of life score for which higher scores represented better quality of life.

Religious coping was the primary independent variable and was measured with the brief RCOPE instrument. The RCOPE was designed to assess how respondents dealt with stressful life situations and consists of two 5-item subscales capturing positive and negative forms of coping.^41^ Positive religious coping questions asked respondents whether they agree “not at all” (coded 0), somewhat (coded 1), “quite a bit” (coded 2), or “a great deal” (coded 3) with the following statements: “I think about how my life is part of a larger spiritual force”; “I work together with God as partners to get through hard times”; “I look to God for strength, support, and guidance in crises”; “I try to find the lesson from God in crises”; and “I confess my sins and ask for God’s forgiveness.” Responses to these items were added together to generate a score. Higher scores were indicative of greater positive religious coping. The negative religious coping questions asked respondents to respond to the following statements: “I feel that stressful situations are God’s way of punishing me for my sins or lack of spirituality”; “I wonder whether God has abandoned me”; “I try to make sense of the situation and decide what to do without relying on God”; “I question whether God really exists”; and “I express anger at God for letting terrible things happen.” The response categories in this domain are reverse coded; therefore “a great deal,” “quite a bit,” “somewhat,” and “not at all” are coded “0,” “1,” “2,” and “3,” respectively. The items for this subscale were summed to generate a negative religious coping score. Higher scores represented greater negative religious coping.

Clinical variables were also included in this analysis. Treatment modality was measured with indicator variables, indicating whether the prostate cancer treatment received by a respondent involved prostatectomy, radiation beam, radiation seeds, hormone therapy, watchful waiting, or another form of treatment. The Gleason score measure in this study was derived from values on pathology reports and classified into low- (Gleason score ≤6), medium- (Gleason score = 7), and high-grade (8 ≥ Gleason score ≤ 10) cancer. The time between diagnosis and treatment variable was a categorical variable classifying responses into “less than 3 months,” “between 3 and 8 months,” and “more than 8 months” categories. Respondents reporting watchful waiting as their treatment had missing data for this variable because this modality did not involve intervention, making the time between diagnosis and initial treatment difficult to report and assess.

Demographic variables were included in this analysis. Self-reported age, measured in years, was included as a continuous variable. Race was represented by a dichotomous variable indicating whether the respondent was black (coded 1) or white (coded 0). Marital status was represented by a dichotomous variable indicating whether respondents reported being married (coded 1) or not (coded 0). The income measure was a categorical variable classified into 3 categories: “less than US$50 000,” “US$50 000 to US$100 000,” and “more than US$100 000.” Educational attainment represented by a categorical variable with 5 categories: “less than high school graduate,” “high school graduate,” “some college or associate’s degree,” “baccalaureate degree,” and “graduate degree.” Health insurance was represented by a dichotomous variable indicating whether respondents reporting having private health insurance, Medicare, Medicaid, CHAMPUS, or CHAMPVA (coded 1) or not (coded 0).

### Data Analysis

Sample characteristics were described using means and standard deviations for continuous variables and proportions for categorical variables; *t* tests and χ^2^ tests were used to present mean and proportional differences between black and white men across all variables, including individual RCOPE components. Multivariable ordinary least squares regression models were estimated to determine the association between religious coping and overall quality of life, adjusting for clinical and demographic covariates. Values of *P* less than .05 were considered significant. All statistical analyses were conducted with StataSE, version 15.

## Results

The description of study participants is displayed for the total sample and by race in Table 1. The analytic sample was almost equally divided between black (N = 306) and white men (N = 316). Black respondents were on average 2 years younger than their white counterparts (61.9 ± 7.7 vs 63.7 ± 7.7) and the proportion of black married men (70.6%) was considerably lower than their white peers (89.0%). Black participants were also less affluent than white sample members. Approximately 1 of every 10 black men in the sample (9.5%) reported incomes over 100 000, while more than one-third of white respondents (34.3%) were in the same category. Slightly over 25% of black sample members earned at least a baccalaureate degree while more than half of their white peer (52.6%) had the same level of educational attainment. The proportion of black participants with health insurance (88.9%) was also smaller than the corresponding percentage of white sample members (96.8%). Black and white men were different on all but 3 variables in the study. On average, black men in the study had lower overall quality of life scores (134.6 ± 19.6) than their white peers (139.8 ± 14.1). Black men in the sample also had higher average positive religious coping scores (12.9 ± 3.3) than white men (10.3 ± 4.5). There was also a significant racial difference in the time to treatment as the results in Table 1 indicate that the proportion of black men in the study having more than 8 months lapse before treatment (10.2%) was more than double the corresponding percentage of their white peers (4.1%).

**Table 1.** Characteristics of African American and White Men in the Diagnosis and Decisions in Prostate Cancer Treatment Outcomes Study.^a^

	Total, n = 624	White men, n = 318	AA men, n = 306	*P* value
African American (%)	49.0			
Age, mean (SD)	62.8 (7.7)	63.7 (7.7)	61.9 (7.7)	.004
Married (%)	80.0	89.0	70.6	<.001
Annual income (%)				<.001
<US$50 000	35.9	19.8	52.6	
US$50 000-US$100 000	42.0	45.9	37.9	
>US$100 000	22.1	34.3	9.5	
Education				<.001
Did not complete high school	11.4	6.3	16.7	
High school graduate	26.2	17.7	35.0	
Some college/associate degree	23.6	24.4	22.9	
Baccalaureate degree	21.1	27.5	14.4	
Graduate degree	17.7	24.1	11.1	
Has health insurance (%)	92.9	96.8	88.9	<.001
Gleason score (%)				
Low-grade cancer	51.0	52.5	49.5	.523
Medium-grade cancer	41.1	39.0	43.3	
High-grade cancer	7.9	8.5	7.2	
Treatment received (%)				.250
Prostectomy	71.7	75.2	67.9	
Radiation beam	12.3	10.3	14.5	
Radiation seeds	3.5	2.9	4.1	
Hormone therapy	1.3	1.0	1.7	
Other treatment	4.3	3.2	5.5	
Watchful waiting	6.8	7.4	6.2	
Time to treat (months) (%)				.016
<3	49.5	50.7	48.1	
3-8	43.6	45.3	41.7	
Over 8	7.0	4.1	10.2	
Positive RCOPE score, mean (SD)	11.6 (4.2)	10.3 (4.5)	12.9 (3.3)	<.001
Negative RCOPE score, mean (SD)	1.1 (1.9)	1.1 (1.6)	1.2 (2.2)	.379
Quality of Life score, mean (SD)	137.2 (17.2)	139.8 (14.1)	134.6 (19.6)	<.001

Abbreviations: AA, African American; SD, standard deviation.

^a^ The proportions presented are the rounded, so they may not add up to 100.

A description of the religious coping items for the total sample and by race is presented in Table 2. There are considerable racial differences among the positive RCOPE components. An overwhelming majority of black men in the study report high levels of agreement with each of the positive RCOPE components while the proportion of white men with the same responses is considerably less. For example, slightly over 8 (81.7%) of every 10 black men in study report a great deal of agreement with the statement that they “seek God for strength, support, and guidance in crises,” while a much smaller segment of white respondents (56.6%) have the same response. Racial distinctions among the negative RCOPE components are less demonstrable as black and white men have similar results on 3 of the 5 components. A small segment of black respondents (8.8%) agreed that stressful situations are God’s punishment; however, this proportion was more than 4 times the percentage of white respondents (1.9%) with the same response. The results in Table 2 also indicate that blacks in the study were less likely to assess situations and make decisions without relying on God than their white peers.

**Table 2.** Individual RCOPE Components for the Total Sample and by Race.

	Total, n = 624	White men, n = 318	AA men, n = 306	*P* value
Positive RCOPE components				
Life is part of a larger spiritual force				>.001
Not at all	12.2	16.4	7.8	
Somewhat	17.5	24.8	9.8	
Quite a bit	13.8	16.7	10.8	
A great deal	56.6	42.1	71.6	
Working with God to get through hard times				>.001
Not at all	5.8	8.8	2.6	
Somewhat	15.2	20.1	10.1	
Quite a bit	13.6	16.0	11.1	
A great deal	65.4	55.0	76.1	
Seek God for strength, support, and guidance in crises				>.001
Not at all	4.7	6.9	2.3	
Somewhat	13.6	19.5	7.5	
Quite a bit	12.8	17.0	8.5	
A great deal	68.9	56.6	81.7	
Seek lessons from God in crises				>.001
Not at all	10.4	16.0	4.6	
Somewhat	15.7	22.3	8.8	
Quite a bit	12.0	12.3	11.8	
A great deal	61.9	49.4	74.8	
Sin confession and seeking forgiveness from God				>.001
Not at all	8.7	13.2	3.9	
Somewhat	12.7	16.7	8.5	
Quite a bit	12.8	13.8	11.8	
A great deal	65.9	56.3	75.8	
Negative RCOPE components				
Stressful situations are God’s punishment				>.001
Not at all	91.0	96.2	85.6	
Somewhat	3.7	1.9	5.6	
Quite a bit	0.8	0.3	1.3	
A great deal	4.5	1.6	7.5	
Wonders about abandonment by God				.366
Not at all	95.8	96.5	95.1	
Somewhat	2.7	1.9	3.6	
Quite a bit	0.2	0.0	0.3	
A great deal	1.3	1.6	1.0	
Assessing situations and making decisions without relying on God				.004
Not at all	69.1	65.1	73.2	
Somewhat	14.3	16.0	12.4	
Quite a bit	4.8	7.6	2.0	
A great deal	11.9	11.3	12.4	
Questions existence of God				.454
Not at all	90.1	89.3	90.9	
Somewhat	5.6	6.9	4.3	
Quite a bit	1.3	1.3	1.3	
A great deal	3.0	2.5	3.6	
Expressing anger at God about bad events				.415
Not at all	93.4	92.8	94.1	
Somewhat	5.1	6.3	3.9	
Quite a bit	0.5	0.3	0.7	
A great deal	1.0	0.6	1.3	

Abbreviations: AA, African American.

The association between religious coping and overall quality of life is displayed in Table 3. Results in the unadjusted model indicate positive religious coping was not associated with overall quality of life, whereas negative religious coping was inversely associated with overall quality of life. Specifically, a 1-point increase in the negative religious coping score was associated with a 1.78-point decrease in the overall quality of life score. In the model adjusted for—race, age, marital status, income, education, insurance status, Gleason Score, treatment received, and time to treatment—a 1-point increase in positive religious coping score was associated with a 0.38-point increase in the overall quality of life score. Also in the same adjusted model, a 1-point increase in the negative religious coping score is associated with a 1.48-point decrease in the overall quality of life score.

**Table 3.** Ordinary Least Squares Regression of Quality of Life and Religious Coping Among African American and White Men With Prostate Cancer.^a^

	Unadjusted	Adjusted
Positive RCOPE score	−0.04 (0.16)	0.38 (0.18)^b^
Negative RCOPE score	−1.78 (0.35)^c^	−1.48 (0.40)^c^
African American race		−1.57 (1.59)
Age		0.19 (0.11)
Married		−0.59 (1.87)
Income		
<50 000		Reference
50 000-100 000		5.88 (1.92)^d^
>100 000		7.82 (2.53)^d^
Education		
Less than high school		Reference
High school graduate		5.39 (2.56)^b^
Some college		5.28 (2.78)
Bachelor degree		8.85 (2.96)^d^
Graduate degree		6.82 (3.15)^b^
Has insurance		3.59 (2.80)
Gleason score		
Low-grade cancer		Reference
Medium-grade cancer		−0.61 (1.46)
High-grade cancer		−4.89 (2.86)
Treatment received		
Prostectomy		Reference
Radiation beam		−0.27 (2.28)
Radiation seeds		5.61 (3.76)
Hormone therapy		−8.18 (6.65)
Other treatment		−1.19 (3.60)
Watchful waiting		−1.30 (4.51)
Time to treat (months)		
<3		Reference
3-8		−0.67 (1.45)
>8		−4.56 (2.80)

^a^ Table entries are unstandardized coefficients.

^b^ *P* < .05.

^c^ *P* < .001.

^d^ *P* < .01.

## Discussion

Prostate cancer is curable (with early detection) and survivable with treatment. The relative 5-year survival rate for black and white men is 97% and 99%, respectively, and the 10-year survival rate for all races and stages combined is 98%.^4^ Quality of life is a salient concern, given the likelihood of extended life after treatment. Surgery, radiotherapy, hormone therapy, and chemotherapy can be disruptive and burdensome in a manner that negatively influences quality of life in addition to overall mental and physical health. Religious coping has been used by individuals to deal with stressful life circumstances including illness and recovery^41^ and our study examined the degree to which this form of coping is associated with quality of life among black and white men with prostate cancer. Both positive and negative forms of religious coping were correlated with quality of life in their expected ways. The degree to which individuals trust in their relationship with God and believe that this relationship will support them during crises was associated with higher quality of life. Positive religious coping was related to enhanced quality of life among men in the study. Negative religious coping had an inverse association with quality of life. The level of distrust in or skepticism about God’s presence was related to lower quality of life. It is noteworthy that both forms of religious coping are statistically significant because the models included social (socioeconomic status) and clinical (type of cancer, treatment received) factors known to have implications for quality of life.

Our findings are consistent with other studies examining religious coping and quality of life in healthy populations^44^ as well as those experience mental and physical challenges.^45-48^ Negative religious coping has been generally associated with lower quality of life; however, there is less consensus about the influence of positive religious coping. We found positive religious coping to be associated with better quality of life while some studies found the relation between this form of coping and quality of life to be nonsignificant.^46^ The present study contributes to this line of work because it provides insight on religious coping among a unique sample—black and white men with prostate cancer. Gender is an often overlooked, yet an important consideration in the faith and health literature. In general, men have lower levels of religious participation than women^49^ and the relative impact of religious coping on their health outcomes remains unclear. Our study presents data indicating that faith-oriented beliefs or perceptions can have implications for quality of life among men with prostate cancer in several and important ways, and future studies are needed to determine the direction and degree to which religious coping is associated with delayed disease onset or progression among men.

Our study also provides some insight about the complexity of race. On average, black men in the sample had higher levels of positive religious coping and lower quality of life scores than their white peers, yet the race variable was not significant in the fully adjusted regression model. It is likely that the racial gap in quality of life scores is a function of the stark socioeconomic status differences between black and white men in the sample. The proportion of affluent and highly educated white sample members more than triples and doubles the corresponding segments of black men in the study respectively. The quality of life benefits afforded by higher incomes and education levels are experienced disproportionately by white patients with prostate cancer in the study. Race and socioeconomic status are often inextricably linked at the individual level,^22,28^ and it is noteworthy that these measures can also serve as proxies for community-level factors (eg, neighborhood disadvantage, access to social and health services) influencing quality of life.^31^ The influence of socioeconomic status on outcomes such as quality of life among black patients with prostate cancer is less clear and merits further understanding.

This study contributes to our understanding of the association between religious coping and quality of life among men with a serious chronic condition; however, there are noteworthy limitations. Religious coping represents only one dimension of religiosity or spirituality and may be serving as a proxy for other factors (eg, church attendance, religious or spiritual social support) potentially influencing quality of life. Data are generated from models estimated using data drawn from a sample of black and white patients with prostate cancer residing in a single Mid-Atlantic state. A substantial segment of the sample report no negative coping and the distribution for positive religious coping is skewed in the opposite direction. These patterns could have analytic implications; however, they suggest that the results may not be generalizable to the larger population of patients with prostate cancer. The analytic models were estimated using cross-sectional data which does not allow causal inferences. A substantial segment of data used in this research are responses to items on an interviewer-administered survey. Indices like the FACT-P are typically included on self-administered questionnaires; therefore, the different modes of data collection could exacerbate social desirability bias.^50^ However, social desirability and recall bias are common in survey research and forced-choice items and validated indices and questions used in well-established surveys were employed to reduce these forms of bias.^50^

Many of the aforementioned limitations could be addressed with a longitudinal study with a richer set of biological, social, economic, religious, and spiritual measures along with a larger sample of black and white men from different areas of the United States. Data from this type of study would allow the investigation of biopsychosocial pathways undergirding associations between religious coping, quality of life, and health outcomes among men with prostate cancer and other health challenges. Investigators of this type would illuminate the clinical significance of statistically significant associations present in our study.

## Conclusions

Prostate cancer remains a significant impediment in the lives of black men and is further exacerbated for many of these men by the presence of stress and a diminished quality of life. Faith, a resource for coping, is seen as a source of comfort and strength across the cancer control spectrum of diagnosis, treatment, and survivorship. When faced with a health crisis like prostate cancer, men need to draw on helpful ways to make meaning of the experience and to see them through. Religion and spirituality may be a bridge to effective coping and should be considered in the context of clinical care. Sensitivity to the role of religion, spirituality, and faith should not be overlooked or stereotyped but seen by providers of health care as potential opportunities for improved outcomes in patients with prostate cancer and survivors.
